# Dermoscopy vs. histopathology in palmoplantar psoriasis and hyperkeratotic eczema: a cross-sectional study

**DOI:** 10.1080/07853890.2026.2749195

**Published:** 2026-11-02

**Authors:** Radha Rathi, Shitij Goel, Shivam Garg, Anshu Gupta Devra

**Affiliations:** a Department of Dermatology, Venereology, and Leprosy School of Medical Sciences and Research, Sharda University, Greater Noida, India; b Department of Pathology, School of Medical Sciences and Research, Sharda University, Greater Noida, India

**Keywords:** Dermoscopy, histopathology, hyperkeratotic palmoplantar lesions, palmoplantar psoriasis, palmoplantar eczema, diagnostic accuracy

## Abstract

**Background:**

Hyperkeratotic palmoplantar lesions (HPPLs), primarily encompassing palmoplantar psoriasis (PPP) and hyperkeratotic palmoplantar eczema (HPKE), present a significant diagnostic challenge due to overlapping clinical morphological features. This study evaluates the diagnostic accuracy of dermoscopy as a non-invasive bridge between clinical evaluation and standard histopathology.

**Methods:**

An observational cross-sectional study was conducted on 44 adult patients presenting with clinically diagnosed HPPLs at a tertiary care center over an 18-month period. Patients underwent thorough clinical examination, dermoscopic evaluation (using a DermLite DL200 Hybrid), and a standard 4.0 mm punch biopsy for histopathological confirmation. Inter-method agreement and diagnostic concordance among the three modalities were analyzed using Cohen’s kappa statistic.

**Results:**

The study cohort (mean age 40.32 years) was histopathologically confirmed to have psoriasis in 31 cases (70.45%) and eczema in 11 cases (25%). Dermoscopically, regularly distributed red dots and globules strongly indicated psoriasis (45.16% vs. 0% in eczema), whereas irregular vascular patterns predominated in eczema (54.55%). Concordance between dermoscopic and histopathological diagnoses was remarkably high, showing good agreement (81.81% concordance, κ = 0.652, *p* < 0.0001). Conversely, clinical-histopathological agreement was poor (56.82%, κ = 0.041), and clinical-dermoscopic agreement was fair (65.90%, κ = 0.350).

**Conclusions:**

The findings of dermoscopy align closely with histopathology. Dermoscopy serves as an effective adjunctive tool to minimize reliance on invasive skin biopsies, rather than completely replacing them.

## 1. Introduction

The skin on the palms and soles possesses a uniquely thickened epidermis, characterized by an extra stratum lucidum and a highly compact stratum corneum. Consequently, diseases affecting this region – particularly hyperkeratotic palmoplantar lesions (HPPLs) – present a common final morphological pathway of hyperkeratosis, lichenification, and fissuring, which often blunts classical clinical signs like vesiculation. Despite involving a small surface area, HPPLs inflict a disproportionately severe burden on patient quality of life, frequently impairing manual dexterity, hindering ambulation, and leading to occupational disability.

In dermatology, HPPLs represent a formidable diagnostic challenge. The primary differential diagnosis lies between Palmoplantar Psoriasis (PPP) and Hyperkeratotic Palmoplantar Eczema (HPKE), alongside other entities like tinea manuum, hypertrophic lichen planus, and acquired palmoplantar keratoderma (PPK) [1]. Differentiating these can be difficult, and overlapping conditions such as ‘eczema in psoriatico’ further complicate clinical evaluation [2]. The traditional diagnostic pathway relies on clinical assessment and, when ambiguous, histopathological examination (HPE). While HPE is the gold standard, it is an invasive procedure associated with pain, potential scarring, risk of infection, and patient reluctance.

Dermoscopy has emerged as a non-invasive, in-vivo imaging technique that bridges clinical dermatology and dermatopathology. Its expanding application in inflammatory dermatoses – termed ‘inflammoscopy’ – allows the visualization of vascular and scale morphological features that are imperceptible to the naked eye, though clinicians must remain aware of potential diagnostic pitfalls [3]. Recent large-cohort studies, such as those by Çetinarslan et al. and Chauhan et al. have successfully defined key morphological characteristics that distinguish palmoplantar psoriasis from hyperkeratotic eczema globally [6,7]. However, a gap remains in understanding how these established dermoscopic clues perform when integrated into the routine, real-world clinical workflow of a tertiary care center in the Indian demographic. Validating these findings within this specific population is crucial for determining if dermoscopy can reliably minimize the need for invasive procedures.

Building upon these prior studies, this cross-sectional study aimed to systematically evaluate the correlation between clinical, dermoscopic, and histopathological findings in patients with HPPLs to establish reliable diagnostic criteria and streamline point-of-care management.

## 2. Materials and methods

### 2.1. Study design and patient selection

An observational cross-sectional study was conducted in the Department of Dermatology, Venereology & Leprosy at a tertiary care center in Greater Noida over an 18-month period starting from *April 2024 to November 2025*. Institutional ethics committee (IEC) approval was taken from IEC, School of Medical Sciences and Research, Sharda University Ref. No. SU/SMS&R/76-A/2024/121 on 08/04/2024. The study was conducted in accordance with the principles of the Declaration of Helsinki.

Sample Size Determination: The sample size was formally calculated based on the outpatient department (OPD) incidence rates of the two primary target diseases: palmoplantar psoriasis ($P_{1}=0.005$) and hyperkeratotic hand-foot eczema ($P_{2}=0.02$). Using the standard formula for estimating proportions with a 95% confidence level (Standard Normal Variation, Z=1.96) and a predefined margin of error of 5% (d=0.05), the minimum required sample size was calculated as N≥38. To ensure adequate statistical power and account for potential exclusions, a total of 44 patients were enrolled in this study.

Forty-four adult patients (aged 21–66 years) presenting with clinically diagnosed hyperkeratotic lesions of the palms and/or soles were enrolled. Written informed consent was obtained from all participants. 1 clinician and 1 pathologist were involved in the study. The clinical and dermoscopic evaluation was performed by the clinician and the reporting dermatopathologist was fully blinded to both the clinical presentation and dermoscopic findings to eliminate interpretation bias.

Exclusion criteria included the application of topical medications within the prior two weeks, systemic therapy within four weeks, infective causes (e.g. HIV, Hepatitis B/C), known bleeding disorders, and hypersensitivity to local anesthetics. KOH mounts were performed initially to rule out dermatophyte infections (Tinea manuum or pedis).

### 2.2. Clinical and dermoscopic evaluation

A detailed clinical history and physical examination were recorded for each patient. Dermoscopic examination of the lesions was performed utilizing a DermLite DL200 Hybrid dermatoscope, featuring 21 LEDs (15 polarized, 7 non-polarized) and 10X magnification with cross-polarization. Images were captured using a Samsung smartphone.

### 2.3. Histopathological examination

A standard 4.0 mm punch biopsy was obtained from a representative lesional site under local anesthesia. Samples were sent for histopathological examination to the Department of Pathology for definitive diagnosis. The evaluating dermatopathologist was blinded to the patients’ clinical presentations and dermoscopic findings. The predefined microscopic criteria utilized to differentiate the lesions were as follows: Psoriasis was characterized by the presence of parakeratosis, supra-papillary thinning, dilated tortuous blood vessels, and Munro’s or Kogoj microabscesses. Eczema was primarily identified by the presence of marked epidermal spongiosis. Lichen planus was defined by wedge-shaped hypergranulosis, basal-layer vacuolar alteration, and a dense band-like lymphocytic infiltrate. Palmoplantar keratoderma was diagnosed based on massive hyperkeratosis and acanthosis without significant inflammation or parakeratosis.

### 2.4. Statistical analysis

Data compilation was performed using Microsoft Excel, and statistical analysis was executed *via* SPSS version 22 (SPSS Inc., Chicago, IL, USA). Categorical variables were reported as frequencies and percentages. To assess the strength of diagnostic agreement between clinical, dermoscopic, and histopathological modalities, inter-method agreement was quantified using Cohen’s kappa statistic. A p-value of ≤ 0.05 was considered statistically significant.

## 3. Results

### 3.1. Demographic and clinical profile

The study cohort consisted of 44 patients with a mean age of 40.32 ± 11.9 years, demonstrating a male preponderance (68.18%). Bilateral symmetrical involvement was the most frequent presentation (63.64%), with lesions most commonly affecting both the palms and soles simultaneously (47.73%). The duration of disease was highly chronic, with 40.91% of patients experiencing symptoms for more than two years (Table 1).

**Table 1.** Duration of disease distribution.

Duration of disease	Frequency	Percentage
0–6 months	11	25.00%
6 months–1 year	5	11.36%
1–2 years	10	22.73%
More than 2 years	18	40.91%
Total	44	100.00%

### 3.2. Final diagnoses

Histopathological examination, serving as the definitive gold standard, confirmed a diagnosis of psoriasis in 31 patients (70.45%), eczema in 11 patients (25.00%), lichen planus in 1 patient (2.27%), and palmoplantar keratoderma in 1 patient (2.27%) (Table 2).

**Table 2.** Histopathological diagnosis distribution.

Histopathological diagnosis	Frequency	Percentage
Psoriasis	31	70.45%
Eczema	11	25.00%
Lichen planus	1	2.27%
Palmo-plantar keratoderma	1	2.27%
Total	44	100.00%

### 3.3. Dermoscopic and histopathological correlation

Dermoscopic analysis identified distinct scale morphologies and vascular patterns distinguishing the primary differential diagnoses (Table 3). Background erythema was uniformly present in all psoriasis cases (100%) and most eczema cases (72.73%). Crucially, regular red dots and globules were observed in 45.16% of psoriasis cases but were entirely absent in eczema, which predominantly displayed irregular red dots and globules (54.55%) (Table 3; Figure 1).

**Figure 1.**
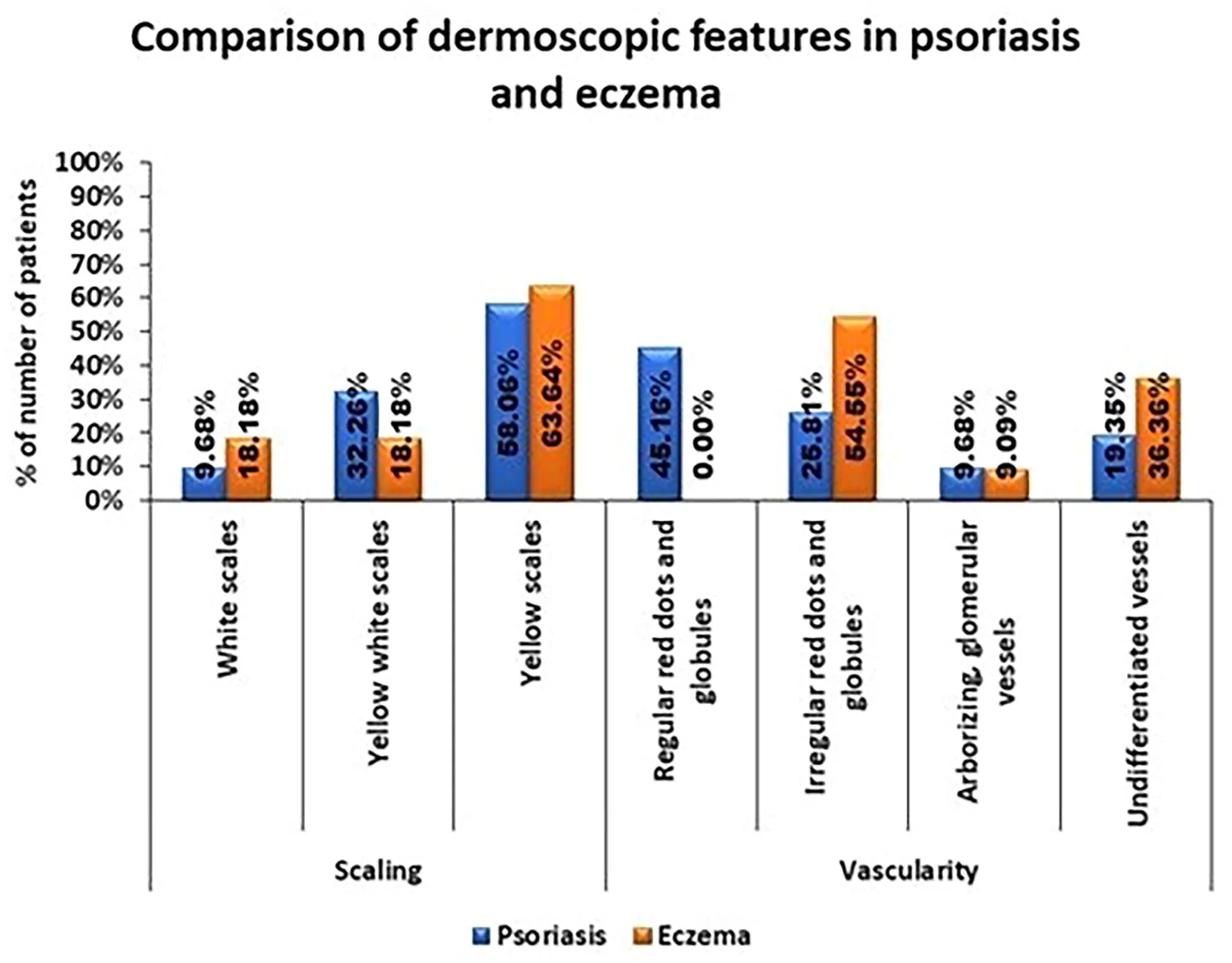
Comparison of dermoscopic features in psoriasis and eczema.

**Table 3.** Distribution of dermoscopic features across all histopathological diagnoses.

Dermoscopic features	Psoriasis (*n* = 31)	Eczema (*n* = 11)	Lichen Planus (*n* = 1)	Palmoplantar keratoderma (*n* = 1)
**Scale morphology**				
White scales	3 (9.68%)	2 (18.18%)	1 (100%)	0 (0%)
Yellow-white scales	10 (32.26%)	2 (18.18%)	0 (0%)	0 (0%)
Yellow scales	18 (58.06%)	7 (63.64%)	0 (0%)	1 (100%)
**Vascular patterns**				
Regular red dots and globules	14 (45.16%)	0 (0%)	0 (0%)	0 (0%)
Irregular red dots and globules	8 (25.81%)	6 (54.55%)	0 (0%)	0 (0%)
Arborizing, glomerular vessels	3 (9.68%)	1 (9.09%)	0 (0%)	0 (0%)
Undifferentiated vessels	6 (19.35%)	4 (36.36%)	0 (0%)	0 (0%)
**Other features**				
Background erythema	31 (100%)	8 (72.73%)	1 (100%)	0 (0%)
Keratotic plugs	0 (0%)	2 (18.18%)	0 (0%)	0 (0%)

Histopathologically, psoriasis was characterized primarily by hyperkeratosis (80.65%), parakeratosis (80.65%), acanthosis (70.97%), dilated tortuous blood vessels (54.84%), and Munro’s microabscesses (48.39%). In contrast, spongiosis was the defining hallmark of eczema, present in 90.91% of cases (Figures 2 and 3). The single case of lichen planus uniquely demonstrated wedge-shaped hypergranulosis, basal-layer vacuolar alteration, and a dense band-like infiltrate (Table 4; Figure 4).

**Figure 2.**
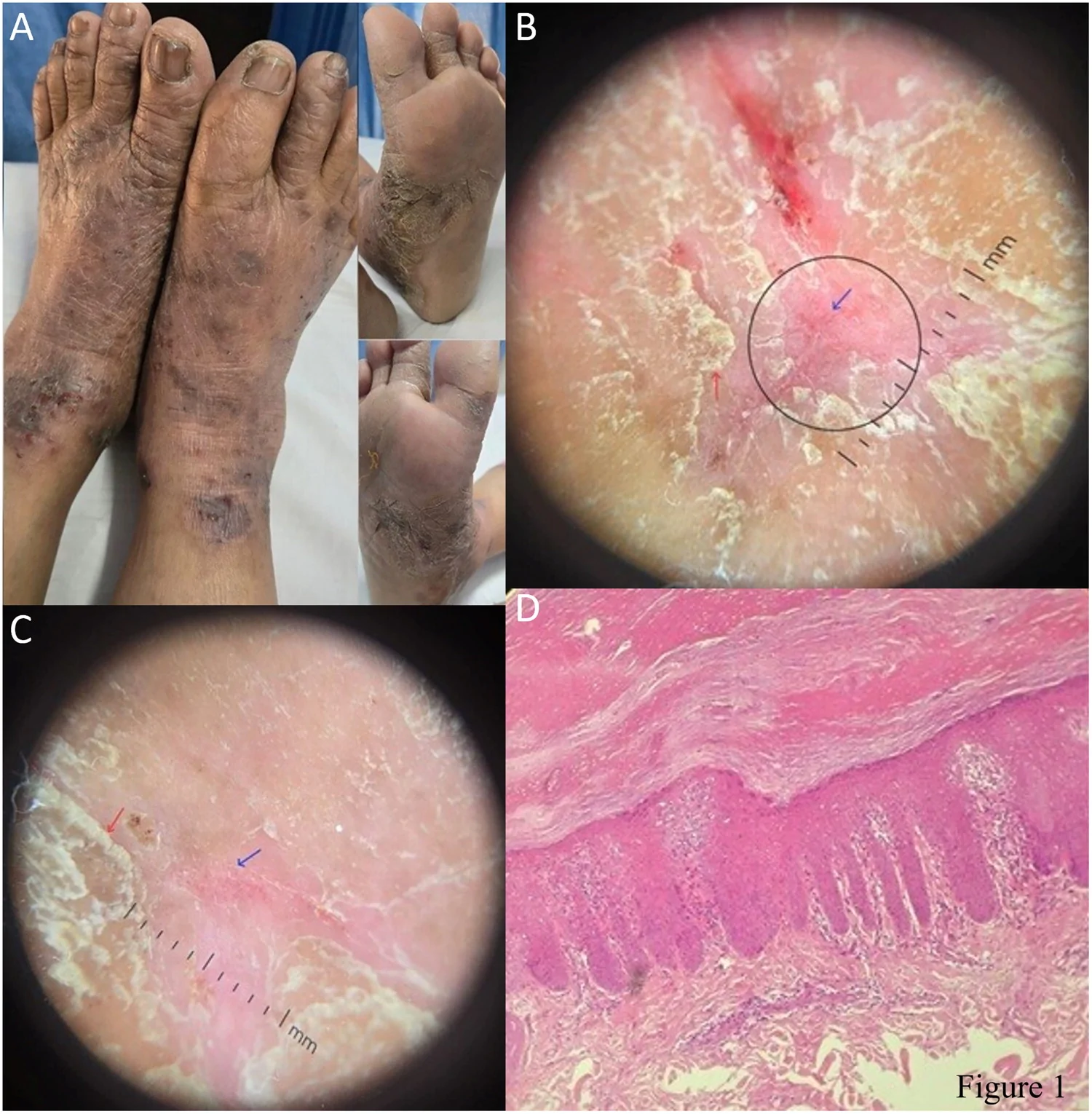
Palmoplantar psoriasis. **(A)** Clinical presentation of bilateral palmoplantar psoriasis. **(B)** Dermoscopy showing yellowish-white scales (red arrow), regularly arranged red dots (blue arrow), and background erythema (black circle) (DermLite DL200 Hybrid). **(C)** Dermoscopy highlighting yellowish-white scales (red arrow) and regularly arranged red dots (blue arrow) (DermLite DL200 Hybrid). **(D)** Histopathology demonstrating parakeratosis, alternating hyper- and hypogranulosis, acanthosis, elongated rete ridges, and a focal Munro’s microabscess with perivascular lymphocytic infiltration (Hematoxylin & Eosin, ×10).

**Figure 3.**
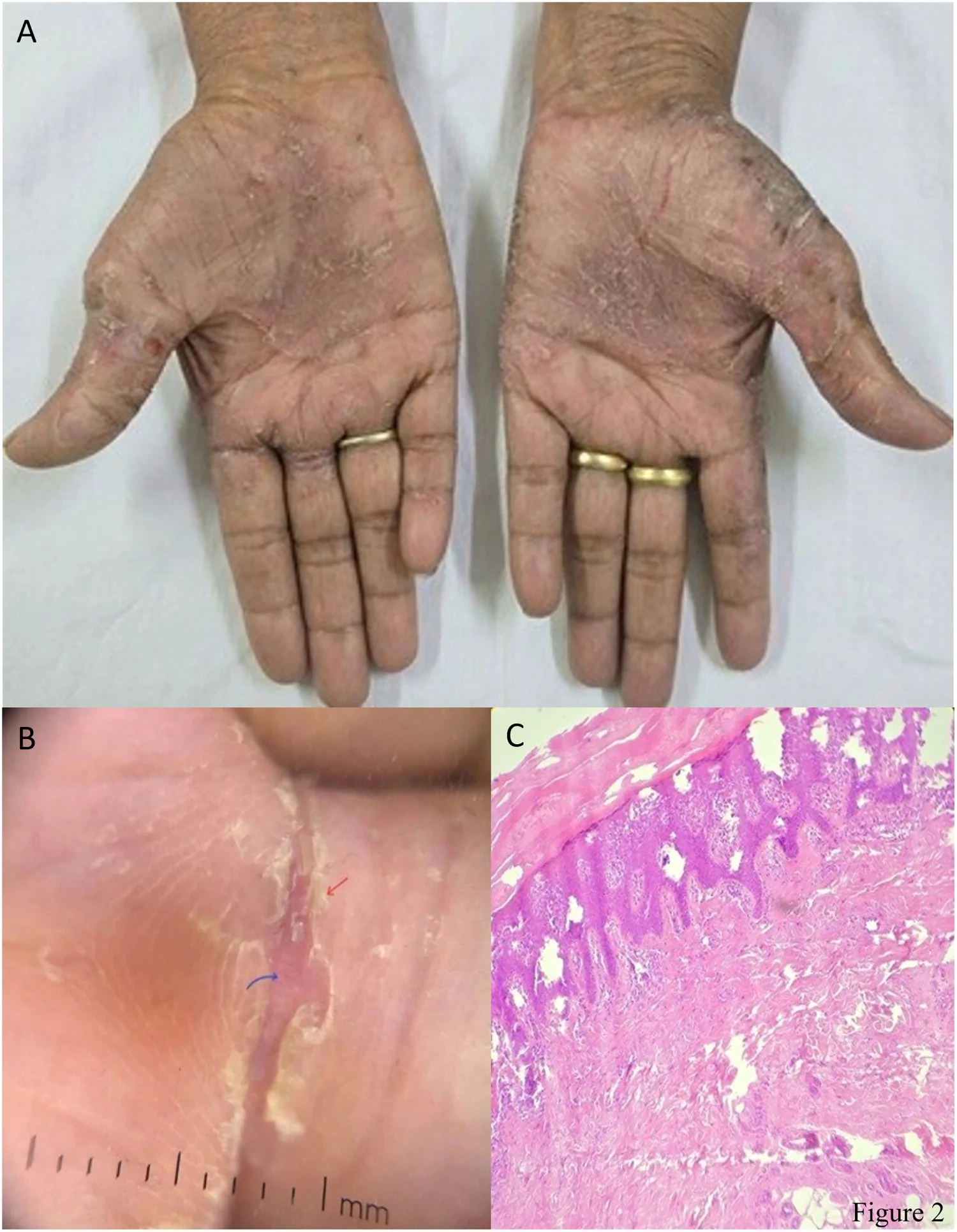
Hyperkeratotic hand eczema. **(A)** Clinical presentation of hyperkeratotic hand eczema. **(B)** Dermoscopy showing yellowish scales (red arrow) and undifferentiated blood vessels (blue arrow) (DermLite DL200 Hybrid). **(C)** Histopathology demonstrating hyperkeratosis, acanthosis, and spongiosis with the formation of intraepidermal vesicles (Hematoxylin & Eosin, ×10).

**Figure 4.**
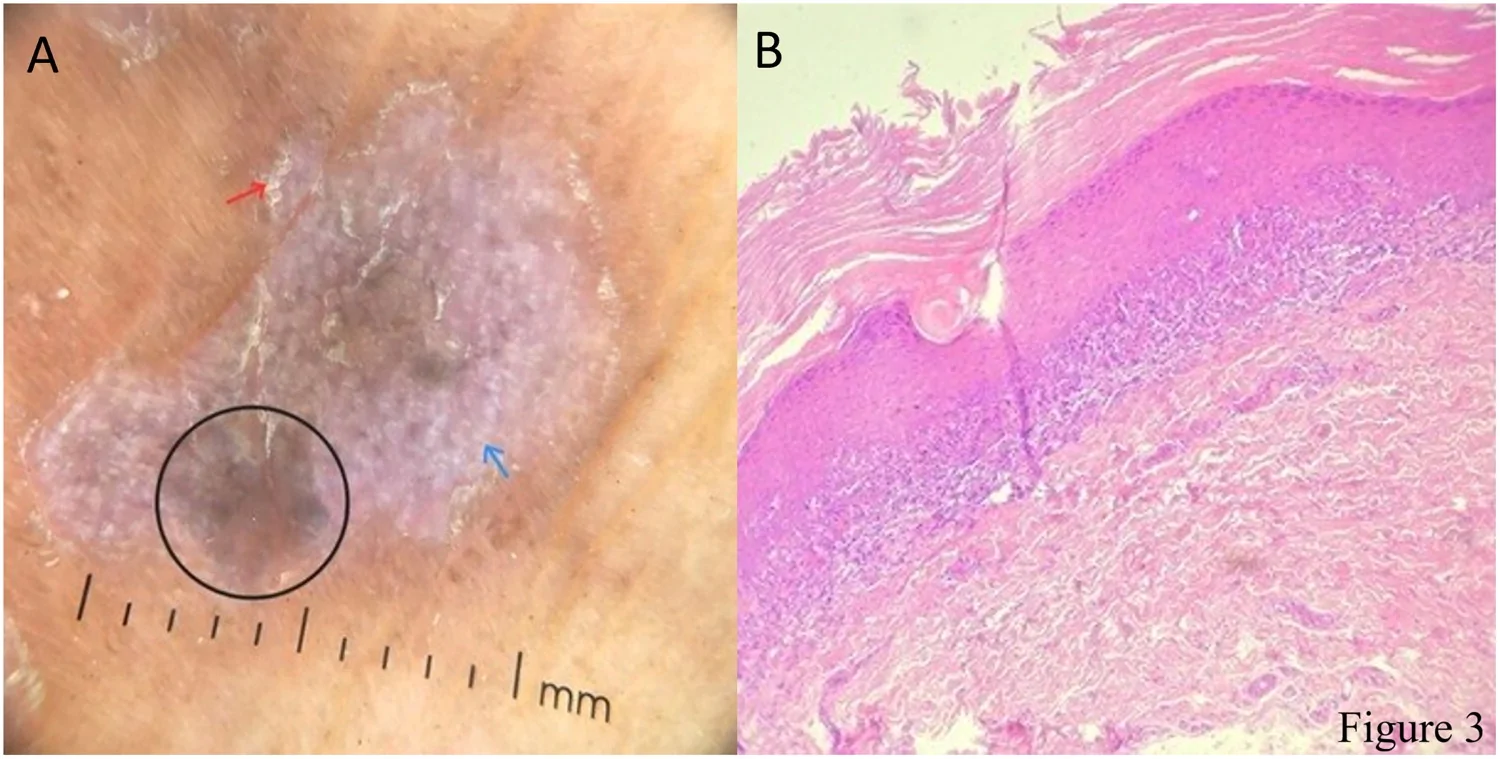
Lichen planus. **(A)** Dermoscopy showing yellow scales (red arrow), Wickham’s striae (blue arrow), and diffuse brown pigmentation on a red background (black circle) (DermLite DL200 Hybrid). **(B)** Histopathology demonstrating mild acanthosis, wedge-shaped hypergranulosis, vacuolar alteration of the basal layer, and a dense band-like lymphocytic infiltrate at the dermo-epidermal junction (Hematoxylin & Eosin, ×10).

**Table 4.** Distribution of histopathological features across diagnostic categories.

Histopathological features	Psoriasis (*n* = 31)	Eczema (*n* = 11)	Lichen planus (*n* = 1)	Palmoplantar keratoderma (*n* = 1)
**Epidermal changes**				
Hyperkeratosis	25 (80.65%)	6 (54.55%)	0 (0%)	1 (100%)
Acanthosis	22 (70.97%)	6 (54.55%)	1 (100%)	1 (100%)
Parakeratosis	25 (80.65%)	3 (27.27%)	0 (0%)	0 (0%)
Spongiosis	10 (32.26%)	10 (90.91%)	0 (0%)	0 (0%)
Supra-papillary thinning	16 (51.61%)	0 (0%)	0 (0%)	0 (0%)
Wedge-shaped hypergranulosis	0 (0%)	0 (0%)	1 (100%)	0 (0%)
Basal-layer vacuolar alteration	0 (0%)	0 (0%)	1 (100%)	0 (0%)
**Dermal changes and infiltrates**				
Munro’s microabscess	15 (48.39%)	0 (0%)	0 (0%)	0 (0%)
Kogoj microabscess	3 (9.68%)	0 (0%)	0 (0%)	0 (0%)
Dilated tortuous blood vessels	17 (54.84%)	0 (0%)	0 (0%)	0 (0%)
Dense band-like infiltrate	0 (0%)	0 (0%)	1 (100%)	0 (0%)

### 3.4. Concordance analysis

A central aim was evaluating the diagnostic accuracy of dermoscopy compared to standard clinical evaluation. Dermoscopy-histopathology agreement demonstrated the strongest diagnostic correlation (81.81% concordance rate, κ = 0.652, *p* < 0.0001), significantly exceeding the poor agreement between clinical evaluation and histopathology (56.82%, κ = 0.041, *p* = 0.750) (Table 5). Specifically, among the 31 histopathologically confirmed psoriasis cases, dermoscopy accurately diagnosed 23 (74.19%), and correctly identified all 11 (100%) of the confirmed eczema cases.

**Table 5.** Diagnostic agreement analysis (kappa statistics).

Diagnostic comparison	Concordance rate	Kappa value	Agreement level
Dermoscopy vs Histopathology	81.81%	0.652	Good ✓
Clinical vs Dermoscopy	65.90%	0.35	Fair
Clinical vs Histopathology	56.82%	0.041	Poor

To further evaluate the diagnostic performance of dermoscopy, diagnostic accuracy metrics were calculated using histopathology as the reference standard (Table 6). For the diagnosis of palmoplantar psoriasis, dermoscopy demonstrated a sensitivity of 74.2% (95% CI: 56.8–86.3) and a specificity of 100.0% (95% CI: 77.2–100.0). The positive predictive value (PPV) and negative predictive value (NPV) were 100.0% and 61.9%, respectively, with an overall diagnostic accuracy of 81.8%.

**Table 6.** Diagnostic accuracy of dermoscopy for palmoplantar psoriasis and eczema compared to histopathology.

Metric	Psoriasis estimate (95% CI)	Eczema estimate (95% CI)
**Sensitivity**	74.2% (56.8–86.3)	100.0% (74.1–100.0)
**Specificity**	100.0% (77.2–100.0)	75.8% (59.0–87.2)
**Positive Predictive Value (PPV)**	100.0% (85.7–100.0)	57.9% (36.3–76.9)
**Negative Predictive Value (NPV)**	61.9% (40.9–79.2)	100.0% (86.7–100.0)
**Overall Accuracy**	81.8% (68.0–90.5)	81.8% (68.0–90.5)

## 4. Discussion

The accurate diagnosis of HPPLs relies heavily on distinguishing between PPP and HPKE, an imperative step due to fundamentally divergent advanced therapeutic algorithms.

Our findings strongly validate the utility of dermoscopy in routine clinical practice. The most critical diagnostic discriminator observed was the vascular architecture. Regularly distributed red dots and globules serve as a direct visual correlate of the underlying psoriatic histopathology – uniform elongation of rete ridges and superficially displaced, dilated papillary capillaries. This feature was highly specific to psoriasis in our cohort (45.16%), aligning with previous dermoscopic studies that emphasize regular vessel arrangement as indicative of palmoplantar psoriasis [4]. Conversely, the chaotic inflammatory process and marked spongiosis characteristic of eczema displace superficial vasculature irregularly, correlating with the irregularly distributed red dots and globules seen in over half (54.55%) of the eczema patients [4].

It is also crucial to acknowledge the histopathological overlap observed in our cohort, which further justifies the use of adjunctive dermoscopy. While spongiosis is the defining hallmark of eczema, present in 90.91% of our eczema cases, it was also observed in a substantial proportion (32.26%) of psoriasis cases. This microscopic overlap can sometimes render relying solely on biopsy challenging and ambiguous. By identifying distinct in-vivo vascular patterns – such as regular versus chaotic red dots and globules – dermoscopy provides an independent, visually objective parameter that helps resolve this histopathological ambiguity, making the differential diagnosis more robust.

The superiority of an integrated diagnostic algorithm is evident in our concordance data. Relying solely on clinical morphological assessment yielded a poor agreement with histopathology (56.82%, κ = 0.041). However, introducing dermoscopy to the diagnostic workflow achieved a good agreement with the gold standard (81.81%, κ = 0.652). This positions dermoscopy as a highly practical instrument capable of stratifying patients into high-probability diagnostic categories, though, as noted in broader clinico-epidemiological literature, it is an adjunctive tool rather than a complete replacement for biopsy in complex presentations [5].

The findings of this study must be interpreted in the context of several limitations. First, the relatively small sample size of 44 patients inherently introduces a risk of statistical bias and restricts the ability to draw broad generalizations, particularly concerning rarer entities like lichen planus and palmoplantar keratoderma. Second, as a single-center study, the results may be influenced by local demographic characteristics and institutional workflows, limiting external validity. Finally, our statistical analysis focused exclusively on inter-method agreement (dermoscopy vs. histopathology) and lacked an assessment of inter-rater variability among different clinicians utilizing the dermatoscope. Future multi-center studies with larger, diverse cohorts are necessary to validate these diagnostic criteria, establish standardized diagnostic thresholds, and systematically evaluate inter-observer reliability across practitioners with varying levels of dermoscopic expertise.

## 5. Conclusion

Dermoscopy significantly enhances diagnostic precision in hyperkeratotic palmoplantar lesions, demonstrating robust concordance with gold-standard histopathological examination. By accurately visualizing disease-specific vascular patterns, dermoscopy provides a highly reliable, non-invasive bridge that outperforms clinical evaluation alone. Dermoscopy acts as a valuable adjunct tool that bridges the gap between clinical suspicion and histopathological confirmation, helping to reduce unnecessary invasive procedures in hyperkeratotic palmoplantar lesions.

## Data Availability

The data supporting the findings of this study are available from the corresponding author upon reasonable request.
